# Exploring the mental health issues of left-behind children based on propensity score matching: the parallel mediating role of psychological abuse and neglect

**DOI:** 10.1186/s13034-025-00992-4

**Published:** 2025-12-09

**Authors:** Cen Lin, Yuqin Song, Lu Pan, Yuhang Wu, Mengqin Dai, Qiuyue Fan, Jiarui Shao, Cailin Xie, Yu Cen, Wenxiu Luo, Jiaming Luo

**Affiliations:** 1 Mental Health Center, Affiliated Hospital of North Sichuan Medical College, No. 1 Maoyuan South Road, Shunqing District, Nanchong City, Sichuan Province China; 2 School of Psychiatry, North Sichuan Medical College, 55 Dongshun Road, Gaoping District, Nanchong City, Sichuan Province China; 3 Key Laboratory of Digital-Intelligent Disease Surveillance and Health Governance, North Sichuan Medical College, 55 Dongshun Road, Gaoping District, Nanchong City, Sichuan Province China

**Keywords:** Psychological abuse, Neglect, Psychological health issue, Parallel mediation model

## Abstract

**Introduction:**

The underdeveloped western region of China, where most of the country’s migrant workers originate, has many children who remain at home while their parents work elsewhere. These left-behind children often suffer from significant psychological issues, including depression, anxiety, stress, and sleep quality problems. The pathways through which being left behind leads to these mental health challenges are not well understood. This study employs Propensity Score Matching (PSM) to adjust for confounders and investigate the mediating effects of psychological abuse and neglect on these mental health issues.

**Methods:**

The study surveyed 17 middle schools in western China using convenience cluster sampling. It gathered data on demographics, the Child Psychological Abuse and Neglect Scale (CPANS), the Depression Anxiety Stress Scale (DASS-21), and the Pittsburgh Sleep Quality Index (PSQI). PSM was used to compare left-behind children with their peers. Statistical tests included Pearson’s chi-squared and bivariate correlation analysis, and mediation was assessed.

**Results:**

A cohort of 12,192 adolescents (mean age = 15.1 ± 1.41 years) was enrolled in this study. Participants with left-behind experiences displayed significantly higher prevalence rates of psychological abuse, neglect, and mental health relative to their non-left-behind counterparts (*p* < 0.05). Following PSM to balance potential confounders, 3,741 matched pairs were successfully yielded. Within these matched cohorts, adolescents with left-behind experiences also demonstrated significantly elevated rates across all measured adverse outcomes (*p* < 0.05). Correlation analysis showed strong associations between left-behind experiences and psychological issues. Mediation analysis revealed that psychological abuse and neglect are significant mediators in the relationship between left-behind experiences and mental health outcomes.

**Conclusion:**

The study suggests that left-behind experiences negatively impact adolescent mental health, acting through mechanisms of psychological abuse and neglect.

## Introduction

 Left-behind children refer to those under the age of 18 who have experienced separation from one or both parents for over six months due to their parents migrating to urban areas for work [1]. The western region of China, which is economically and socially underdeveloped, is one of the country’s largest sources of migrant workers. Many adults leave their children behind in their hometowns for work, often in the care of a single parent, grandparents, or others, leading to a large number of left-behind children in this region [2].

Previous studies have shown that left-behind children are more likely to experience mental health problems [2]. Compared to non-left-behind children, those who are left at home may exhibit more behavioral problems and communication difficulties [3], lower self-esteem and well-being [4], higher tendencies toward academic and social anxiety, as well as physical symptoms [5], all of which increase the risks of depression and suicidal ideation [6] and raise the likelihood of sleep disorders [7]. A 2018 study on the mental health of left-behind children in China indicated a mental health problem prevalence of 43.4% [8], and a 2019 Chinese meta-analysis found that the incidence of mental health problems among left-behind children (8%) was about 2.7 times that of non-left-behind children (3%) [5], Furthermore, a 2018 global meta-analysis revealed that compared to non-left-behind children, left-behind children face a 25% higher risk of depression and an 85% higher risk of anxiety [6], and a 2021 study conducted in Nepal similarly suggests that left-behind children are at a higher risk of experiencing adverse mental health conditions [9].

Psychological abuse involves caregivers failing to provide an appropriate and supportive environment, which negatively impacts the emotional health and development of the child. These behaviors may include restricting the child’s actions, belittling, mocking, threatening, intimidating, discriminating, rejecting, and other non-physical forms of hostile treatment [10]. Neglect refers to the failure of parents, despite being capable, to assist in one or more areas crucial for the child’s development, such as health, education, emotional growth, nutrition, housing, and safe living conditions [10]. Compared to physical abuse, sexual abuse, and physical neglect, psychological abuse, and neglect show no obvious external signs. Second, there are significant regional and cultural differences in defining psychological abuse and neglect [9, 11]. For instance, several researchers have emphasized that “abuse” is a notably severe descriptor. Although such behavior can be detrimental to children, it should not be prematurely classified as child abuse without clear evidence of intent to cause harm [9, 11, 12]. Additionally, Chinese families generally show widespread neglect of children’s mental health issues [13], which has, to some extent, led to less attention from society and relevant organizations regarding psychological abuse and neglect.

Empirical studies have identified the left-behind phenomenon—a developmental experience characterized by parent-child separation—as a causal determinant of multiple adverse outcomes. These include heightened risks of child health impairments [14], behavioral and emotional disorders [15], and non-suicidal self-injury (NSSI) [16], among other psychosocial sequelae. Although parents may provide enhanced financial support for children and adolescents, their absence frequently precipitates deficiencies in familial caregiving, emotional support, and affective communication [17]. Consequently, left-behind children exhibit heightened vulnerability to emotional neglect compared to their non-left-behind peers [18].​ Secondarily, parental absence compels grandparents to assume primary caregiving responsibilities for most left-behind children. The significant intergenerational disparities in age and cultural values between these groups structurally impede effective communication [19]. Moreover, certain traditional Chinese educational philosophies actively endorse authoritarian disciplinary approaches [19], predisposing caregivers to disproportionately employ stringent child-rearing practices. This cultural predisposition consequently elevates the risk of psychological abuse and neglect [20].​ Furthermore, psychological abuse and neglect demonstrate robust associations with severe psychological disturbances, including anxiety [21, 22], depression [22, 23], post-traumatic stress disorder (PTSD) [22], psychiatric disorders [24], attention deficits [25], substance use disorders [25], and sleep disturbances [26]. However, psychological abuse and neglect are closely associated with severe mental health issues such as anxiety [21, 22], depression [22, 23], PTSD [22], mental illness [24], attention deficit [25], as well as substance addiction [25] and sleep disorders [26].

Nevertheless, few studies have explored the mechanisms linking the mental health problems of left-behind children. The following areas are key in these discussions: first, previous studies mentioned the attachment theory, which posits that long-term, high-quality companionship, care, and strong emotional investment are essential to maintaining parent-child attachment relationships [27]. However, the prolonged separation between left-behind children and their parents reduces parent-child interaction [28, 29], damages attachment relationships, increases children’s vulnerability, and triggers emotional distress, such as anxiety, depression, and anger. These negative effects tend to worsen over time [20]. On the other hand, prior research has also introduced the family resilience theory, which suggests that adolescents living in positively functioning families are more likely to communicate actively and positively when facing crises or challenges, thereby helping them solve problems, boost confidence and security, and reduce negative emotions and psychological distress [30]. However, parental absence alters family functioning, weakens emotional bonds, and leaves adolescents less capable of problem-solving and adapting to their environment when facing stress and challenges, thereby increasing their risk of mental health problems [31].

Most previous studies on the adverse mental health outcomes among left-behind children were small-scale cross-sectional studies, often focused on economically developed and easily accessible cities, with limited variables included and potential confounding biases [5, 8]. Moreover, previous research commonly used conventional simple mean comparison methods (such as *t*-tests and ANOVA), which are less effective in controlling for confounding factors and may lead to bias [5, 8].

Given this context, this study aims to match two groups, left-behind and non-left-behind children, using the Propensity Score Matching (PSM) method. PSM calculates the probability of participants being assigned to the left-behind children group through a logit model (this value is the propensity score) and matches each participant in the left-behind group with a non-left-behind participant with a similar propensity score, thereby balancing potential confounders between the two groups [32]. The following hypotheses will be tested: **H1**: Relative to their non-left-behind counterparts, left-behind children manifest significantly heightened levels of psychological abuse, neglect, and mental health-issues (depression/anxiety/stress/sleep quality). **H2**: Left-behind experiences demonstrated significant positive correlations with psychological abuse, neglect, and mental health problems. **H3**: Psychological abuse and neglect mediate the relationship between left-behind experiences and mental health problems.

### Survey and inclusion criteria

This study is a multi-center cross-sectional study aimed at evaluating adolescent mental health. A two-sided test was required with an alpha level of 0.05 and a permissible error (δ) of 1. The latest survey data in China indicates that the prevalence of mental health problems among left-behind children is approximately 43.4% [8]. Using PASS 15 software, the minimum sample size was calculated to be 7,103 cases. Given the potential causes of data loss, including incomplete questionnaire responses and clear errors or misentries, we increased the sample size by 20% based on prior studies [33], raising the minimum sample size to 8,524 cases.

Survey Methods: From November 2021 to May 2022, convenience cluster sampling was used to select three counties from three cities in western China. A total of 17 middle schools participated, and 14,210 students aged 12 to 18 years were included in the questionnaire survey. The questionnaire was distributed online through WJX (Wenjuanxing, website: https://www.wjx.cn). Before completing the questionnaire, both participants and their parents were informed of the study’s purpose. After obtaining informed consent from parents and students, the questionnaire was completed.

Quality Control: The data were collected in two phases. In the first phase, the researchers were required to familiarize themselves with the study procedures and the content of the questionnaire, receiving training on how to properly complete it. They also communicated the study’s purpose and content with the headteachers of the participating classes and participants’ parents, and after reaching a consensus, the questionnaires were distributed uniformly. Following obtaining informed consent, researchers administered QR code-accessible questionnaires requiring completion within a demarcated timeframe. Adolescents independently completed all surveys without parental interference. In the second phase, two researchers collected the completed questionnaires and checked for data accuracy, exporting the results from the WJX platform to an Excel spreadsheet. Prior to data analysis, missing values, obvious outliers, and inconsistent responses were removed, and the remaining data that met the inclusion criteria were analyzed, such as data entry aberrations (e.g., biologically implausible values such as age = 180 years) or systematic response contradictions and non-credible answers, indicating participant inattentiveness, comprehension deficits, or random responding. The research team consists of practicing physicians from the Psychiatric and Psychological Department and graduate students specializing in psychiatry at the same institution. Before the survey, they underwent systematic training to familiarize themselves with the procedures, content, and subsequent processes, and all researchers strictly followed Standard Operating Procedures (SOPs).

### Data collection and measurement

#### Sociodemographic data

The demographic information collected in this study includes age, gender, grade, left-behind experience, living environment, father’s education, mother’s education, father’s occupation, mother’s occupation, and whether the child lives in a boarding school. In the self-compiled demographic questionnaire, the concept and definition of left-behind children were explained in detail. After a thorough explanation by trained personnel and teachers, children identified their own status based on their experiences and completed the questionnaire.

#### Child psychological abuse and neglect scale (CPANS)

The CPANS was developed by Deng Yunlong et al., and is a retrospective measurement tool suitable for use in the Chinese population [34]. It primarily assesses experiences of psychological abuse and neglect during childhood (under 18 years of age), including family abuse, neglect, and caregiver treatment. The scale consists of 31 items and is divided into two subscales: psychological abuse and neglect. The psychological abuse subscale includes factors such as scolding, intimidation, and interference, with items like “My parents listed all my faults in front of others” and “My parents verbally abused me unexpectedly.” The neglect subscale includes emotional, educational, and physical/supervisory neglect, with items like “My parents did not care about my academic performance” and “My parents did not stop me from drinking alcohol.” The scale uses a 5-point Likert scoring method, with higher total scores indicating more severe psychological abuse and neglect experienced during childhood. A mean score of ≥ 1 on the psychological abuse subscale indicates the presence of psychological abuse, while a mean score of ≥ 1 on the neglect subscale indicates the presence of neglect. The internal consistency reliability (Cronbach’s α) of the total CPANS scale is 0.858, with the psychological abuse subscale at 0.794 and the neglect subscale at 0.820. The internal consistency reliability of the three dimensions of psychological abuse ranges from 0.555 to 0.637, and for the three dimensions of neglect, it ranges from 0.547 to 0.686. In this study, Cronbach’s α for CPANS was 0.921, with the internal consistency reliability for the psychological abuse and neglect dimensions ranging from 0.756 to 0.830 and from 0.698 to 0.803, respectively. The validity of the scale was 0.971. Permission for the use of this scale was granted by its authors.

#### The depression anxiety stress scale (DASS-21)

Developed by Lovibond et al. in 1995, the DASS-21 is used to distinguish and define common emotional disorders such as depression, anxiety, and stress [35]. It provides auxiliary psychometric indicators for clinical diagnosis and offers a fast and effective tool for participant screening in research. The simplified Chinese version of the DASS-21 was revised by Wen Yi et al., in 2012 for use in the Chinese population [36]. The scale is divided into three subscales for depression, anxiety, and stress, with each subscale containing 7 items, for a total of 21 items. Scores are classified into five severity levels: normal, mild, moderate, severe, and extremely severe, corresponding to 1–5 points. In this study, the Cronbach’s α coefficient was 0.948.

#### The Pittsburgh sleep quality index (PSQI)

The PSQI was developed by Buysse and his team in 1989 to assess sleep quality over the past month [37]. The scale contains 24 items, including 19 self-rated questions and 5 clinician-rated questions, but the 19th self-rated question and all 5 clinician-rated questions are not scored, leaving a total of 18 scored items. These items form seven dimensions: sleep onset latency, sleep duration, sleep quality, sleep efficiency, sleep disturbances, use of sleep medications, and daytime dysfunction. Each dimension is scored on a scale from 0 to 3, with the total score ranging from 0 to 21. Higher scores indicate poorer sleep quality. In this study, the Cronbach’s α coefficient for the PSQI was 0.868.

### Statistical analysis

**Fig. 1:**
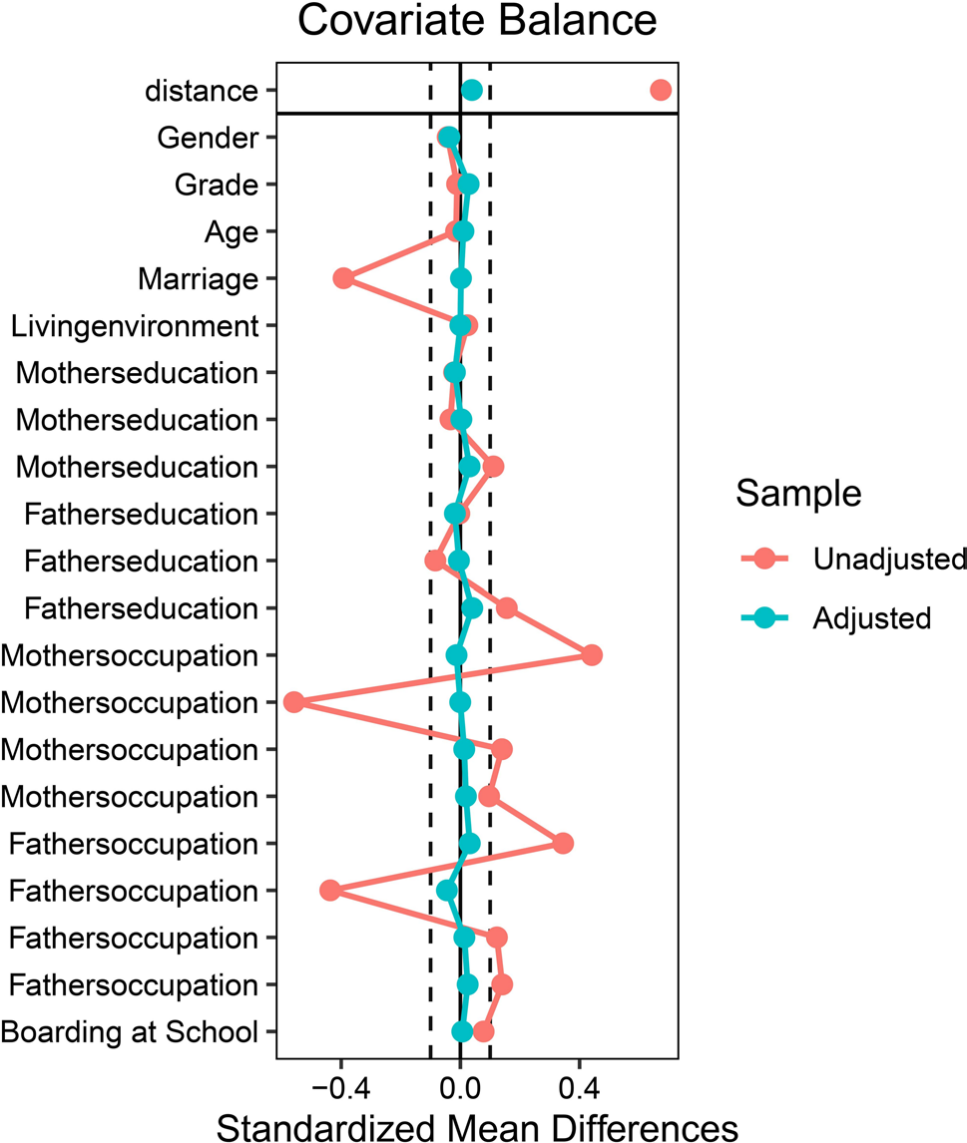
SMD point diagram

**Fig. 2:**
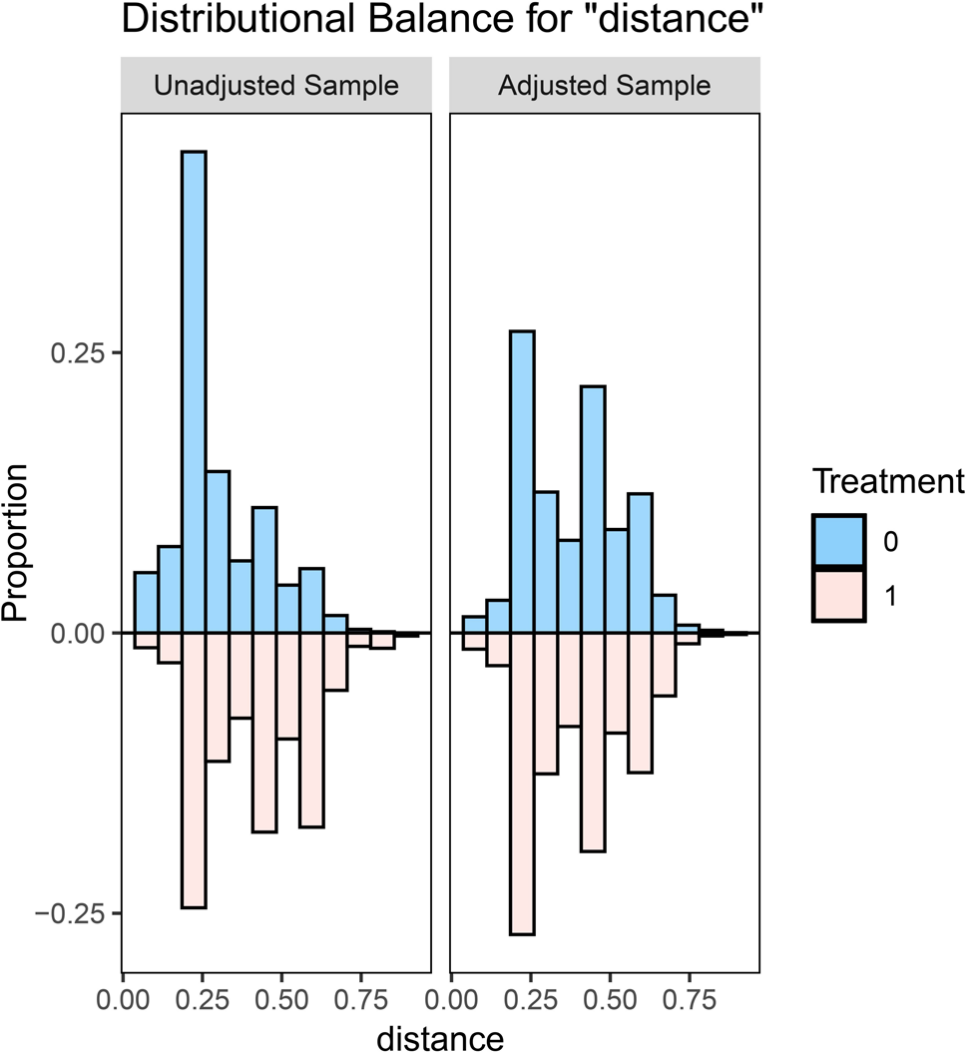
Image histogram

First, descriptive statistics were performed using SPSS 26.0 to describe the demographic characteristics, psychological abuse, neglect, and mental health issues (depression, anxiety, stress, and sleep quality) of left-behind and non-left-behind children before PSM matching. Categorical variables were presented as numbers and percentages. The Pearson chi-squared test was used to assess the statistical differences between left-behind and non-left-behind children, with two-tailed p-values less than 0.05 considered statistically significant. Second, to eliminate potential confounding factors and address selection bias that might affect the study results, we matched the groups using gender, parental marital status, living environment, parental education, parental occupation, and whether the child lived in a boarding school as covariates. PSM matching between the two groups was performed using the R 4.2.2 software packages “MatchIt,” “tableone,” and “cobalt“ [38]. The logit function was selected for the matching model, with the caliper value set to 0.2*SD [39]. Nearest neighbor matching without replacement was performed in a 1:1 ratio. The standardized mean difference (SMD) was used to assess the balance of demographic characteristics after matching, with an absolute value of SMD less than 0.1 considered indicative of a good matching balance [40, 41]. The balance of confounding factors before and after matching was displayed using a dot-line plot for SMD (Fig. 1), and a mirrored histogram was used to show the distribution of propensity scores for left-behind and non-left-behind children before and after matching (Fig. 2). Third, descriptive statistics using SPSS 26.0 were again conducted to describe the differences in demographic characteristics, psychological abuse, neglect, and mental health problems between left-behind and non-left-behind children after PSM. Categorical variables were represented as numbers and percentages, and statistical significance between the two groups was assessed using Pearson’s chi-squared test, with two-tailed p-values less than 0.05 considered statistically significant. Fourth, bivariate correlation analysis was conducted on the matched sample to examine the correlation between left-behind experiences, psychological abuse, neglect, and mental health issues. Fifth, Mediation analysis was carried out using the SPSS PROCESS version 4.0 developed by Hayes. PROCESS Model 4 was employed as a parallel mediation model to explore whether psychological abuse/neglect mediates the relationship between left-behind experiences and depression, anxiety, stress, and sleep quality. This study followed the recommendations of Hayes and Preacher [42], using the Bootstrap method with 5,000 samples to analyze different mediation effects of the independent variables. Bootstrapping was chosen because it allows us to avoid Type I errors that might arise from the non-normal distribution of indirect effects. A 95% confidence interval (CI) for indirect effects, with lower limits (LL) and upper limits (UL) not including zero, indicated a significant mediation effect.

## Results

A total of 14,210 questionnaires were collected in this survey, of which 12,192 were valid, and 2,018 were invalid (due to missing data, obviously fabricated responses, or inconsistent answers), resulting in an effective questionnaire rate of 86% (Fig. 1).

### Demographic characteristics

Among the 12,192 adolescents, the participants’ ages ranged from 12 to 18 years, with an average age of 15.1 ± 1.41 years. Adolescents with left-behind experiences accounted for 66.3% of the sample. Those with left-behind experiences had significantly higher proportions of psychological abuse, neglect, depression, anxiety, stress, and poor sleep quality than adolescents without left-behind experiences. Table 1 shows that there were demographic differences between the two groups (*p* < 0.05). Using the PSM matching method, we successfully matched 3741 pairs (left-behind and non-left-behind children). After matching, the covariate scores were all below 0.1, as shown in Table 1. Figure 1 demonstrates the balance of covariates, before matching, revealing significant pretreatment differences (standardized mean differences, SMDs > 0.1) between treatment and control groups across covariates including parental marital status, residential environment, paternal education, and parental employment, whereas post-matching standardized mean differences were < 0.1 with nonsignificant intergroup disparities (*p* > 0.05). Figure 2 illustrates that the symmetry of propensity scores between the two groups improved significantly after matching, reducing sample bias and enhancing the similarity of the two sample groups, indicating good balance. After balancing potential confounding factors, adolescents with left-behind experiences continued to show higher proportions of psychological abuse, neglect, depression, anxiety, stress, and poor sleep quality than their non-left-behind peers (*p* < 0.05) (see Table 1).

**Table 1:** Differences in demographic characteristics before PSM matching

	Mode	Pre-PSM demographic characteristics				
		Total	Left-behind experience		X²	p(Bilateral)
		12,192	Yes	No		
Age		15.1 ± 1.40	15.1 ± 1.42	15.0 ± 1.35		
Gender	Female	5690 (46.7%)	3832 (67.3%)	1858 (32.7%)	4.93	0.03
	Male	6502 (53.3%)	4255 (65.4%)	2247 (34.6%)		
Grade	Junior High	6989 (57.3%)	4621 (66.1%)	2368 (33.9%)	0.33	0.57
	Senior High	5203 (42.7%)	3466 (66.6%)	1737 (33.4%)		
Parental Marital Status	At marriage	10,392 (85.2%)	6607 (63.6%)	3785 (36.4%)	238.81	0.00
	Divorced	1800 (14.8%)	1480 (82.2%)	320 (17.8%)		
Living Environment	Urban	3467 (28.4%)	1961 (56.6%)	1506 (43.4%)	207	0.00
	Rural	8725 (71.6%)	6126 (70.2%)	2599 (29.8%)		
Father’s Education	Elementary School	3928 (32.2%)	2610 (66.4%)	1318 (33.6%)	99.96	0.00
	Middle School	7604 (62.4%)	5156 (67.8%)	2448 (32.2%)		
	College and Above	660 (5.4%)	321 (48.6%)	339 (51.4%)		
Mother’s Education	Elementary School	5249 (43.1%)	3510 (66.9%)	1739 (33.1%)	45.99	0.00
	Middle School	6392 (52.4%)	4285 (67.0%)	2107 (33.0%)		
	College and Above	551 (4.5%)	292 (53.0%)	259 (47.0%)		
Father’s Occupation	Farming	2006 (16.5%)	916 (45.7%)	1090 (54.3%)	717.95	0.00
	Migrant Worker	8927 (73.2%)	6506 (72.9%)	2421 (27.1%)		
	Business owner	1107 (9.1%)	627 (56.6%)	480 (43.4%)		
	Civil Servant	152 (1.2%)	38 (25.0%)	114 (75.0%)		
Mother’s Occupation	Farming	3358 (27.5%)	1634 (48.7%)	1724 (51.3%)	915.53	0.00
	Migrant Worker	7555 (62.0%)	5765 (76.3%)	1790 (23.7%)		
	Business owner	1172 (9.6%)	651 (55.5%)	521 (44.5%)		
	Civil Servant	107 (0.9%)	37 (34.6%)	70 (65.4%)		
Boarding at School	Yes	7085 (58.1%)	4805 (67.8%)	2280 (32.2%)	16.79	0.00
	No	5107 (41.9%)	3282 (64.3%)	1825 (35.7%)		
Psychological Abuse	Present	4766 (39.1%)	3409 (71.5%)	1357 (28.5%)	94.63	0.00
	Absent	7426 (60.9%)	4678 (63.0%)	2748 (37.0%)		
Neglect	Present	3989 (32.7%)	2844 (71.3%)	1145 (28.7%)	65.46	0.00
	Absent	8203 (67.3%)	5243 (63.9%)	2960 (36.1%)		
Sleep Quality	Good	8247 (67.7%)	5288 (64.1%)	2959 (35.9%)	57.82	0.00
	Fair	3287 (27.0%)	2327 (70.8%)	960 (29.2%)		
	Average	586 (4.8%)	425 (72.5%)	161 (27.5%)		
	Poor	67 (0.5%)	42 (62.7%)	25 (37.3%)		
Depression	Present	3338 (27.4%)	2384 (71.4%)	954 (28.6%)	53.31	0.00
	Absent	8854 (72.6%)	5703 (64.4%)	3151 (35.6%)		
Anxiety	Present	5182 (42.5%)	3672 (63.1%)	1510 (36.9%)	82.82	0.00
	Absent	7010 (57.5%)	4415 (55.8%)	2595 (44.2%)		
Stress	Present	2057 (16.9%)	1482 (72.0%)	575 (28.0%)	36.21	0.00
	Absent	10,135 (83.1%)	6605 (65.2%)	3530 (34.8%)		

### Bivariate correlation analysis

As shown in Table 2, the bivariate correlation analysis of the matched sample revealed significant positive correlations between left-behind experiences and psychological abuse, neglect, depression, anxiety, stress, and poor sleep quality. Significant positive correlations were also found between psychological abuse and neglect, depression, anxiety, stress, and poor sleep quality (see Table 2).

**Table 2:** Differences in demographic characteristics after PSM matching

	Mode	Total	Left-behind experience		X²	*p*(Bilateral)
		7482	Yes	No		
Age		15.0 ± 1.37	15.0 ± 1.38	15.0 ± 1.36		
Gender	Female	3277 (43.8%)	1673 (51.1%)	1604 (48.9%)	2.59	0.11
	Male	4205 (56.2%)	2068 (49.2%)	2137 (50.8%)		
Grade	Junior High	4321 (57.8%)	2186 (50.6%)	2135 (49.4%)	1.43	0.23
	Senior High	3161 (42.2%)	1555 (49.2%)	1606 (50.8%)		
Parental Marital Status	At marriage	634 (8.5%)	3425 (50.0%)	3423 (50.0%)	0.01	0.93
	Divorced	6848 (91.5%)	316 (49.8%)	318 (50.2%)		
Living Environment	Urban	2100 (28.1%)	1050 (50.0%)	1050 (50.0%)	0.00	1.00
	Rural	5382 (71.9%)	2691 (50.0%)	2691 (50.0%)		
Father’s Education	Elementary School	2362 (31.6%)	1197 (50.7%)	1165 (49.3%)	3.79	0.15
	Middle School	4617 (61.7%)	2313 (50.1%)	2304 (49.9%)		
	College and Above	503 (6.7%)	231 (45.9%)	272 (54.1%)		
Mother’s Education	Elementary School	3060 (40.9%)	1547 (50.6%)	1513 (49.4%)	2.39	0.30
	Middle School	4030 (53.9%)	2012 (49.9%)	2018 (50.1%)		
	College and Above	392 (5.2%)	182 (46.4%)	210 (53.6%)		
Father’s Occupation	Farming	1546 (20.7%)	747 (48.3%)	799 (51.7%)	5.9	0.12
	Migrant Worker	4913 (65.7%)	2498 (50.8%)	2415 (49.2%)		
	Business owner	932 (12.5%)	458 (49.1%)	474 (50.9%)		
	Civil Servant	91 (1.2%)	38 (41.8%)	53 (58.2%)		
Mother’s Occupation	Farming	2860 (38.2%)	1442 (50.4%)	1418 (49.6%)	1.74	0.63
	Migrant Worker	3559 (47.6%)	1780 (50.0%)	1779 (50.0%)		
	Business owner	1000 (13.4%)	492 (49.2%)	508 (50.8%)		
	Civil Servant	63 (0.8%)	27 (42.9%)	36 (57.1%)		
Boarding at School	Yes	3456 (46.2%)	2019 (50.1%)	2007 (49.9%)	0.08	0.78
	No	4026 (53.8%)	1722 (49.8%)	1734 (50.2%)		
Psychological Abuse	Present	1668(36.5%)	921 (55.2%)	747 (44.8%)	28.59	0.00
	Absent	2900(63.5%)	1363 (47.0%)	1537 (53.0%)		
Neglect	Present	1551(34.0%)	854 (55.1%)	697 (44.9%)	24.06	0.00
	Absent	3017(66.0%)	1430 (47.4%)	1587 (52.6%)		
Sleep Quality	Good	3087(67.6%)	1480 (47.9%)	1607 (52.1%)	18.44	0.00
	Fair	1175(25.7%)	626 (53.3%)	549 (46.7%)		
	Average	268(5.9%)	156 (58.2%)	112 (41.8%)		
	Poor	38(0.8%)	22 (57.9%)	16 (42.1%)		
Depression	Present	1163(25.5%)	640 (51.7%)	523 (45.0%)	15.79	0.00
	Absent	3405(74.5%)	1644 (48.3%)	1761 (55.0%)		
Anxiety	Present	1789(39.2%)	974 (52.9%)	815 (45.6%)	23.23	0.00
	Absent	2779(60.8%)	1310 (47.1%)	1469 (54.4%)		
Stress	Present	1781(39.0%)	967 (54.3%)	35.6% (45.7%)	21.54	0.00
	Absent	2787(61.0%)	1317 (47.3%)	1470 (52.7%)		

### Parallel mediation model

**Fig. 3:**
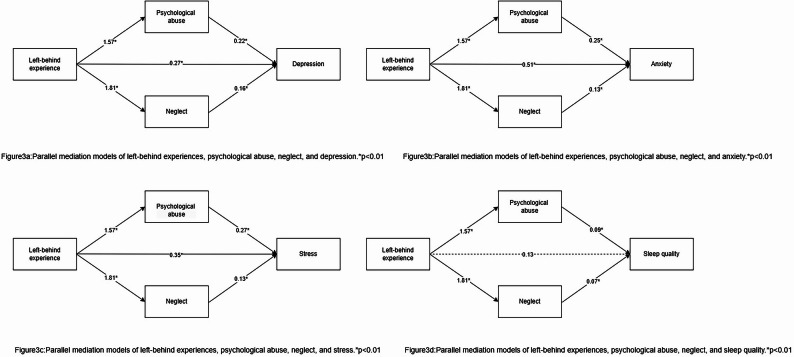
Parallel mediator model. **a** Parallel mediation models of left-behind experiences, psychological abuse, neglect, and depression. *p<0.01.** b** Parallel mediation models of left-behind experiences, psychological abuse, neglect, and anxiety. *p < 0.01.** c** Parallel mediation models of left-behind experiences, psychological abuse, neglect, and strees. *p < 0.01.** d** Parallel mediation models of left-behind experiences, psychological abuse, neglect, and sleep quality. *p < 0.01

Using the Model 4 parallel mediation model, the total effects of left-behind experiences on depression, anxiety, and stress were 0.91 [0.60, 1.21], 1.13 [0.83, 1.43], and 1.00 [0.67, 1.33], When psychological maltreatment and neglect were included in the analysis, the direct effects of left-behind experience on depression, anxiety, and stress decreased to 0.27 [95% CI 0.02, 0.52], 0.51 [95% CI 0.26, 0.76], and 0.35 [95% CI 0.07, 0.62], respectively. All 95% confidence intervals excluded zero (*P* < 0.001). This finding supports the statistical significance of indirect effects through mediating variables, with total effects exceeding direct effects, suggesting the presence of underlying indirect pathways in the research model. The total indirect effects of left-behind experience on depression, anxiety, and stress via psychological abuse and neglect were 0.64 [95% CI 0.46, 0.81], 0.62 [95% CI 0.45, 0.79], and 0.66 [95% CI 0.47, 0.85], respectively. The specific indirect effects attributable to psychological abuse were 0.34 [95% CI 0.24, 0.45], 0.39 [95% CI 0.27, 0.51], and 0.43 [95% CI 0.30, 0.56] for depression, anxiety, and stress, respectively. The specific indirect effects attributable to neglect were 0.29 [95% CI 0.20, 0.39], 0.23 [95% CI 0.16, 0.31], and 0.23 [95% CI 0.15, 0.31] for depression, anxiety, and stress, respectively. All 95% confidence intervals excluded zero (*p* < 0.001), indicating statistically significant indirect paths. Therefore, psychological abuse/neglect partially mediated the relationship between left-behind experience and depression/anxiety/stress. Furthermore, we found that the total effect of left-behind experience on sleep quality was 0.39 [95% CI 0.24, 0.53]. However, when psychological abuse and neglect were controlled for, the direct effect of left-behind experience on sleep quality became non-significant (as the confidence interval included zero, *p* > 0.001). Furthermore, the indirect paths through which left-behind experience affects sleep quality via psychological abuse and neglect were statistically significant (as the confidence intervals excluded zero, *p* < 0.001). The specific indirect effects were 0.04 [95% CI 0.03, 0.06] and 0.04 [95% CI 0.03, 0.05], respectively, accounting for 35% and 33% of the total effect, respectively. Therefore, the results indicate that psychological abuse/neglect fully mediated the relationship between left-behind experience and sleep quality. The specific values for the total effect, direct effect, and proportion mediated are presented in Table 3, and a schematic summary of the research model is provided in Fig. 3.

**Table 3:** Correlation analysis of left-behind experience, psychological abuse, neglect, depression, anxiety, stress, and sleep quality

Parameter	Left-behind experience	Psychological abuse	Neglect	Depression	Anxiety	Stress	Sleep quality
Left-behind experience	1	0.08*	0.08*	0.07*	0.08*	0.07*	0.05*
Psychological abuse		1	0.77*	0.54*	0.55*	0.54*	0.41*
Total neglect score			1	0.53*	0.51*	0.50*	0.41*
Depression				1	0.81*	0.80*	0.50*
Anxiety					1	0.82*	0.51*
Stress						1	0.46*
Sleep quality							1

The table shows the correlations between the variables of left-behind experience, psychological abuse, neglect, depression, anxiety, stress, and sleep quality. *p < 0 0.01

**Table 4:** Parallel mediation effect analysis

Pathway	Effect value		Effect	BootSE	BootLLCI	BootULCI	Effect proportion	*p*
Left-behind Experience → Depression	Total Effect	Total	0.91	0.16	0.60	1.21		0.00
Left-behind Experience → Depression	Direct Effect		0.27	0.13	0.02	0.52	30%	0.00
Left-behind Experience → Psychological Abuse/Neglect → Depression	Indirect Effect	Total Indirect Effect	0.63	0.09	0.46	0.81	70%	0.00
Left-behind Experience → Psychological Abuse → Depression		Psychological Abuse	0.34	0.06	0.24	0.45	38%	0.00
Left-behind Experience → Neglect → Depression		Neglect	0.29	0.05	0.20	0.39	32%	0.00
Left-behind Experience → Anxiety	Total Effect	Total	1.13	0.15	0.83	1.43		0.00
Left-behind Experience → Anxiety	Direct Effect		0.51	0.13	0.26	0.76	45%	0.00
Left-behind Experience → Psychological Abuse/Neglect → Anxiety	Indirect Effect	Total Indirect Effect	0.62	0.09	0.45	0.79	55%	0.00
Left-behind Experience → Psychological Abuse → Anxiety		Psychological Abuse	0.39	0.06	0.27	0.51	34%	0.00
Left-behind Experience → Neglect → Anxiety		Neglect	0.23	0.04	0.16	0.31	20%	0.00
Left-behind Experience → Stress	Total Effect	Total	1.00	0.17	0.67	1.33		0.00
Left-behind Experience → Stress	Direct Effect		0.35	0.14	0.07	0.62	35%	0.00
Left-behind Experience → Psychological Abuse/Neglect → Stress	Indirect Effect	Total Indirect Effect	0.66	0.09	0.47	0.85	65%	0.00
Left-behind Experience → Psychological Abuse→ Stress		Psychological Abuse	0.43	0.07	0.30	0.56	43%	0.00
Left-behind Experience → Neglect → Stress		Neglect	0.23	0.04	0.15	0.31	23%	0.00
Left-behind Experience → Sleep Quality	Total Effect	Total	0.39	0.07	0.24	0.53		0.00
Left-behind Experience → Sleep Quality	Direct Effect		0.13	0.06	−0.00	0.25	32%	0.00
Left-behind Experience → Psychological Abuse/Neglect → Sleep Quality	Indirect Effect	Total Indirect Effect	0.26	0.04	0.19	0.34	68%	0.00
Left-behind Experience → Psychological Abuse → Sleep Quality		Psychological Abuse	0.04	0.01	0.03	0.06	35%	0.00
Left-behind Experience → Neglect → Sleep Quality		Neglect	0.04	0.01	0.03	0.05	33%	0.00

*p < 0.01

## Discussion

This study innovatively applied PSM to match adolescents with and without left-behind experiences from a multi-center sample in western China, effectively eliminating potential confounding factors among included variables. It then explored the mediating role of psychological abuse/neglect in the relationship between left-behind experiences and mental health issues (depression, anxiety, stress, and sleep quality). The study found that compared to non-left-behind children, left-behind children were more likely to experience psychological abuse/neglect and mental health problems. There was a positive correlation between left-behind experiences, psychological abuse/neglect, and poor mental health outcomes. The mediation model revealed a partial mediating effect of psychological abuse/neglect in the relationship between left-behind experiences and depression/anxiety/stress, indicating that left-behind experiences both indirectly elevate risks of depression, anxiety, and stress through psychological abuse and neglect while also exerting direct effects on these outcomes. The mediation model further indicated full mediation by psychological abuse/neglect in the relationship between left-behind experiences and sleep disturbances, signifying that left-behind experiences exclusively elevate risks of sleep disorders through psychological abuse and neglect (See Table 4).

This study identified left-behind experiences as inducing psychological abuse and neglect, aligning with prior empirical evidence [5, 6, 43, 44]. This may be related to weakened family environments, as parental migration alters family structure and function, leaving caregivers, often grandparents, with increased childcare and household burdens. These caregivers often have lower educational levels and poorer mental health, which may prevent them from providing high-quality parenting [45, 46], leading to weakened family environments [45]. Left-behind children growing up in these environments are less likely to have their safety and basic care needs met [44], increasing their risk of psychological abuse and neglect. Some studies also reference the Ecological Model, which emphasizes the interaction between individuals and their environment [47]. Left-behind experiences disrupt parenting, undermining the ecological model and leading to psychological abuse and neglect.

Psychological abuse/neglect leads to adverse mental health issues in adolescents, such as depression, anxiety, stress, and sleep disturbances, a finding consistent with previous research [48]. Earlier studies provide biological explanations, suggesting that early psychological abuse and neglect can physiologically alter neurobiological development, negatively affecting children’s physical, cognitive, emotional, and social growth, leading to long-term psychological and behavioral problems [49, 50]. Moreover, the cognitive coping strategy theory proposes that individuals should adaptively modify their cognition and behavior to cope with stressors [51]. The quality of the parent-child relationship plays a crucial role in adolescents’ ability to develop coping strategies [52]. Children who experience psychological abuse are more likely to form maladaptive cognitive and interpersonal characteristics, which increase psychological vulnerability and the likelihood of developing avoidant coping strategies [53]. Consequently, their risk of mental health problems also increases.

Our study proposes that psychological abuse/neglect mediates the relationship between left-behind experiences and mental health problems. As established previously, left-behind experience serves as a significant predictor of psychological abuse/neglect, which in turn functions as a primary mechanism contributing to mental health issues. We hypothesize that this could be linked to several theories. First, the absence of parents leads to lower-quality parent-child relationships, resulting in psychological abuse and neglect. Children lacking strong social support often experience low self-esteem and psychological resilience [54], making them more susceptible to physical and psychological symptoms when facing negative life events. Additionally, left-behind experiences reduce children’s access to social resources (interpersonal, family support, and developmental resources), limiting their ability to acquire healthy interpersonal skills, such as managing peer relationships [55]. This may lead to increased loneliness, lower well-being, and a higher likelihood of developing psychological and behavioral issues, such as social anxiety and elevated psychological stress [55]. Furthermore, parental absence often forces left-behind children to face academic challenges alone [8]. Besides not providing timely support, parents may place high expectations on their children’s academic performance, believing that academic success will improve the family’s future [8]. These high expectations, coupled with low levels of support, impose psychological pressure on children, increasing the risk of mental health issues, such as academic anxiety [8].

The strength of this study lies in its use of PSM to eliminate confounding factors while analyzing the mediating effects of psychological abuse/neglect between left-behind experiences and mental health outcomes such as depression, anxiety, stress, and sleep quality. This is the first study to propose that psychological abuse/neglect mediates the relationship between left-behind experiences and these mental health issues. Additionally, the study utilizes a large, multi-center dataset, which enhances the generalizability of the theoretical findings. However, there are several limitations to this research. First, all variables were measured through self-reported questionnaires, which may introduce recall bias. Second, the study follows a cross-sectional design, limiting the ability to examine mediation effects over time, as all variables were measured at a single point. Future research should adopt longitudinal designs to explore the long-term effects of left-behind experiences on psychological abuse/neglect and mental health outcomes such as depression, anxiety, stress, and sleep quality. Third, while this study separately investigates the mediating effects of psychological abuse and neglect, it does not account for the frequency or cumulative effects of abuse. For example, it does not consider how many instances of abuse a child experienced or whether psychological neglect occurred alongside abuse. Future studies should explore the effects of multiple or cumulative abuse. Fourth, the study focuses exclusively on left-behind children in China, with data collected during the COVID-19 pandemic, which limits its applicability to the general population or other countries. Fifth, this study did not assess the potential further impact of parental migration duration on the psychological well-being of left-behind children; additionally, it failed to address influences exerted by non-parental caregivers or examine potential associations between improvements in household socioeconomic status and child mental health outcomes. Sixth, as participants were predominantly minors with ongoing cognitive and expressive development, accurately retrospectively reporting complex emotions or long-term experiences proved challenging, potentially leading to oversimplified accounts or omission of critical information; Furthermore, children’s self-identification as “left-behind” may be influenced by personal perceptions and stigmatization, deviating from the study’s operational definition. Lastly, this investigation was notably limited to variables including depression, anxiety, stress, and sleep quality; future research should incorporate broader mental health constructs and implement methodological refinements such as multi-informant reporting and longitudinal assessments.

Notwithstanding these limitations, this study substantially advances the mechanistic understanding of how psychological abuse and neglect mediate the relationships between left-behind experiences and adverse mental health outcomes—specifically depression, anxiety, stress, and sleep disturbances. These findings underscore the imperative for evidence-informed policy interventions. To promote healthy development among left-behind children, governments should enact multifaceted approaches: Primarily, policymakers must reform social policies and establish comprehensive legal frameworks that mandate legal penalties for guardians demonstrating negligence or willful maltreatment, while simultaneously institutionalizing long-term follow-up mechanisms. Subsequently, systematic surveillance of parental abuse/neglect manifestations among left-behind children, integrated with concurrent parental psychological assessments, would enable early identification of latent risks and facilitate targeted interventions. Complementarily, governments should deploy qualified professionals to deliver domiciliary mental health support services, thereby enhancing parental competencies in child development science, developmentally appropriate childcare, and evidence-based parenting methodologies. At the micro-level, educational institutions and familial units fulfill essential functions in safeguarding the psychological well-being of left-behind children. Schools should systematically integrate developmentally appropriate mental health curricula while collaborating with psychiatric specialists to implement evidence-based interventions. For example, these professionals can deliver standardized mental health seminars and public awareness initiatives across multi-system environments—spanning household, educational, and community settings—to propagate preventative mental health literacy. This socioecological approach collectively cultivates supportive pedagogical environments, with integrated implementation demonstrably enhancing living conditions in target regions, concurrently reducing incidence rates of psychological maltreatment and neglect while optimizing developmental trajectories among left-behind children.

## Conclusion

Left-behind experiences lead to a higher risk of mental health issues in adolescents, such as depression, anxiety, stress, and poor sleep quality, with psychological abuse and neglect acting as mediators. This study provides theoretical support for improving the mental health outcomes of left-behind children. By enhancing the living conditions of left-behind children and formulating supportive policies, their healthy development can be promoted to a certain extent.

## Data Availability

The data of this paper is collected by the author on campus, and has a complete database. As the database is related to mental health variables and concerns personal privacy, it will not be made public. Relevant information can be obtained from the author of this article on request.
